# Polyphenol Content and Antioxidant Activity in Homemade and Commercial Soups: A Theoretical and Experimental Approach

**DOI:** 10.3390/antiox14050563

**Published:** 2025-05-08

**Authors:** Monika Sejbuk, Iwona Mirończuk-Chodakowska, Małgorzata Kuczyńska, Anna Maria Witkowska

**Affiliations:** Department of Food Biotechnology, Medical University of Bialystok, Szpitalna 37, 15-295 Bialystok, Poland; iwona.mironczuk-chodakowska@umb.edu.pl (I.M.-C.); m.kuczynska21@gmail.com (M.K.)

**Keywords:** polyphenols, antioxidant activity, processed soups, FRAP assay, electrochemical analysis, theoretical calculation, thermal processing

## Abstract

**Background**: Most studies on polyphenols and antioxidant activity focus on raw ingredients, often overlooking the impact of technological processes—a gap that is particularly notable given that many population studies rely on theoretical calculations from nutritional databases. Therefore, it is essential to verify whether these theoretical values align with experimental findings on model dishes and to determine the extent to which processing affects polyphenol content and antioxidant activity in processed foods. **Methods**: As model dishes, this study analyzed soups prepared through thermal processing, along with commercially available ready-to-eat and instant soups. Total polyphenol content was measured using the Singleton–Rossi method, while antioxidant activity was assessed using the FRAP (ferric-reducing antioxidant potential) method and an electrochemical method. Theoretical calculations were performed based on original recipes from Polish nutritional value tables, as well as data from available polyphenol and antioxidant activity databases for raw ingredients. **Results**: The total polyphenol content varied significantly between experimental measurements and theoretical calculations, with deviations ranging from −42% to +1370%. FRAP antioxidant activity also differed, ranging from −62% to +524%, depending on the type of soup. The polyphenol content in homemade soups ranged from 3.692 to 16.534 mg GAE/100 mL, in ready-to-eat soups from 4.387 to 18.431 mg GAE/100 mL, and in instant soups from 1.624 to 7.254 mg GAE/100 mL, with tomato soups consistently having the highest polyphenol content across all categories. FRAP values ranged from 0.021 to 0.189 mmol/100 g in homemade soups, 0.029 to 0.269 mmol/100 g in ready-to-eat soups, and 0.033 to 0.134 mmol/100 g in instant soups, with tomato soups again showing the highest FRAP values. Antioxidant activity measured electrochemically ranged from 44.410 to 52.467 mC/g in homemade soups, 22.750 to 58.900 mC/g in ready-to-eat soups, and 22.515 to 47.680 mC/g in instant soups, with broccoli soups showing the highest values. **Conclusions**: This study demonstrates that theoretical models alone are insufficient for accurately determining polyphenol content and antioxidant activity in food, reinforcing the importance of experimental validation in processed food.

## 1. Introduction

Non-communicable diseases (NCDs), including cardiovascular diseases, cancer, respiratory illnesses, and diabetes, are responsible for 74% of all deaths globally [1]. Oxidative stress is a major contributor to these diseases, arising from an imbalance between prooxidants and antioxidant defenses, which leads to chronic inflammation and cellular damage [2,3,4,5]. Long-term oxidative stress can trigger preneoplastic changes, apoptosis, and tissue dysfunction. Diet is a major modifiable factor: Western-style diets, high in processed foods, saturated fats, and sugars, increase oxidative stress, while plant-based diets rich in polyphenols and antioxidants may support the antioxidant defense system and reduce inflammation [3,4,5,6,7,8,9].

Polyphenols, abundant in vegetables, fruits, legumes, nuts, seeds, and spices, are potent dietary antioxidants [9]. Their content and activity, however, can be altered by technological processing such as boiling, steaming, or drying [10,11,12,13]. While processing may increase polyphenol bioavailability through matrix softening, it may also lead to the degradation of thermolabile compounds.

Current databases, while comprehensive, predominantly focus on raw ingredients and often overlook the impact of technological processing. The values provided in these databases are typically derived using the Folin–Ciocalteu and FRAP (ferric-reducing antioxidant potential) methods [14,15,16]. Given this context, the present study employs soups—widely consumed, diverse, and highly processed food products—as a representative model to evaluate the influence of processing on polyphenol content and antioxidants activity. Accordingly, this study aims to (1) assess the consistency between theoretical estimates and experimentally determined values of polyphenol content, measured using the Folin–Ciocalteu method, and antioxidant activity, measured using the FRAP assay, in homemade soups; (2) compare these values with commercially available ready-to-eat and instant soups; and (3) investigate the applicability of a novel electrochemical method in conjunction with established analytical techniques to improve the completeness and accuracy of food composition databases.

## 2. Materials and Methods

The study material included three categories of soups: (1) homemade soups, (2) commercially available ready-to-eat soups, and (3) instant soups. A total of 17 types of homemade soups were prepared in triplicate (n = 51 samples). Ready-to-eat soups (n = 14) and instant soups (n = 9) were analyzed in varying numbers of replicates depending on product availability, as indicated in the respective result tables. All commercial samples, as well as the food products used to prepare the homemade soups, were purchased from various retailers to ensure diversity in product selection and to reflect the range of processed and raw food items available to consumers.

### 2.1. Methods

Homemade soups were prepared in the laboratory using standardized recipes based on the Polish Food Composition and Nutritional Value Tables [17]. All ingredients were weighed with precision and cooked without the addition of extra water, using only prepared vegetable- or meat-based broth. Soups were cooked in boiling liquid (95–98 °C) for approximately 20 min, until vegetables were softened. Each soup type was prepared in triplicate. The final product was weighed to determine yield and calculate antioxidant values per 100 g of fresh product.

Ready-to-eat and instant soups were commercially available products purchased from various retail chains available in Poland. Ready-to-eat soups were analyzed as sold, without further modification.

After preparation, all homemade and ready-to-eat soups were frozen using a blast freezer and then lyophilized until constant weight using a LyoQuest 55 lyophilizer, Telstar, Spain. The lyophilized samples were ground using a laboratory electric mill. Instant soups were assayed directly and all values were recalculated to correspond to the ready-to-eat form in accordance with the manufacturer’s instructions.

Methanol–water extracts were prepared from the lyophilized samples and the instant soup samples. A 0.5 g portion of the analyzed material was weighed, then 10 mL of a methanol–water mixture (90/10 *v*/*v*) was added, and the sample was mixed for 3 h on a rotary shaker at a speed of 150 rpm at room temperature. The sample was then left in the dark for 20 h, followed by centrifugation for 10 min at 3000 rpm. The obtained supernatant was used for further analyses.

### 2.2. Determination of Total Polyphenol Content

The total polyphenol content was determined spectrophotometrically according to Singleton and Rossi [18]. This method is based on the colorimetric reaction between phenols and the Folin–Ciocalteau reagent, which contains tungstic acid and molybdic acid. The absorbance of the blue-colored complex was measured at a wavelength of 765 nm. The results were expressed in mmol of Gallic Acid Equivalents (GAEs) per 100 g of sample.

### 2.3. Determination of Antioxidant Activity by the FRAP Method

Total antioxidant activity was assessed using the FRAP (ferric-reducing ability of plasma) method as described by Benzie and Strain [19]. This method is based on the reduction of iron (III) ions, present in a ferric-2,4,6-tris(2-pyridyl)-s-triazine (TPTZ) complex, to iron (II) ions by the antioxidants found in the sample. The reaction produces a blue coloration with an absorption maximum at 593 nm, which was measured after 4 min. The results were expressed in mmol of iron (II) per 100 g of sample.

### 2.4. Determination of Antioxidant Activity Using Electrochemical Method

Antioxidant activity was measured using the electrochemical method with the eBQC device, Bioquochem, Spain. This device measures the resistance of the sample to oxidation, which correlates with its antioxidant capacity. The measurement involves the transfer of electrons from antioxidant compounds to the electrochemical sensor, allowing for a direct and rapid assessment of antioxidant potential. For analysis, extracts were prepared from the soup samples using the standardized extraction procedure described above to ensure consistency. The electrochemical measurements were initially performed on these extracts, and the obtained values were subsequently recalculated to reflect the antioxidant activity in the final product. The antioxidant activity was recorded as the total charge transferred during the process, expressed in millicoulombs per gram of product (mC/g).

### 2.5. Calculation of Polyphenol Content and Antioxidant Activity According to Available Databases

For comparative purposes with experimental data, theoretical values of polyphenol content and antioxidant activity (FRAP) were calculated using food composition databases [15,20]. All calculations were based on the same standardized soup recipes used in the experimental study. The proportions of each ingredient were derived from the Polish Food Composition and Nutritional Value Tables [17], and theoretical values were calculated per 100 g of final product, accounting for yield.

FRAP values for individual ingredients (in mmol/100 g) were primarily obtained from The Antioxidant Food Table by Carlsen et al. [15]. For polyphenol content, data were additionally verified using the Phenol-Explorer 3.0 database [20], when available. All values referred to raw, unprocessed ingredients, consistent with how antioxidant content is typically presented in food composition databases.

Theoretical values for each soup were calculated as weighted sums: the antioxidant value of each ingredient (per 100 g) was multiplied by its percentage share in the recipe (as shown in Supplementary Table S1). Final theoretical polyphenol and FRAP values were expressed in the same units as the experimental results, allowing direct comparison.

To compare the experimental and theoretical values of polyphenol content and antioxidant activity in homemade soups, a Sign test was performed. Statistical significance was considered at *p* < 0.05. All statistical analyses were conducted using Statistica software, version 13.3 (StatSoft, Tulsa, OK, USA).

### 2.6. Statistical Analysis

The normality of data distribution was tested using the Shapiro–Wilk test. Since most values did not follow a normal distribution, the tables include both means with standard deviations and medians with ranges for comparative purposes. Pearson correlation analysis was performed to assess relationships between experimental and calculated values for polyphenol content and FRAP, as well as between polyphenols, FRAP, and electrochemical antioxidant activity in homemade soups. Statistical calculations were conducted using Statistica 13.0 (Tibco, Palo Alto, CA, USA).

K-Means clustering grouped soups by polyphenol content and FRAP antioxidant potential, with the Elbow Method and Silhouette Score determining the optimal number of clusters. Hierarchical clustering (Ward’s method, Euclidean distance) was applied to polyphenol content, FRAP, and electrochemical activity, with dendrograms visualizing the grouping patterns. Additionally, the Sign test was employed to assess the presence of systematic bias between experimental and theoretical estimates of polyphenol content and antioxidant activity. Analyses were performed in Python 3.8 using scipy, pandas, matplotlib, and scikit-learn.

## 3. Results

### 3.1. Polyphenol Content of Homemade Soups

In Table 1, the results of polyphenol content in homemade soups are presented, expressed in mg GAE/100 mL along with the respective calculated values. Among the analyzed homemade soups, tomato cream soup and button mushroom cream soup had the highest average polyphenol content, 16.534 and 15.628 mg GAE/100 mL, respectively. The lowest median polyphenol content was found in pea soup: 3.692 mg GAE/100 mL.

Comparing the results obtained from experimental and calculation methods revealed a wide range of differences, varying from −42% to +1350%. The smallest differences (not exceeding 10%) were observed in mushroom soups, including button mushroom, cream of mushroom, and wild mushroom soups. The largest discrepancy between experimental and calculated values was found in green pea cream soup, with a difference of +1350%. The Sign test showed a predominance of positive differences; however, the result did not reach statistical significance (*p* = 0.072). Nevertheless, the observed trend suggests that the predictive model tended to overestimate polyphenol content compared to experimental measurements.

**Figure 1:**
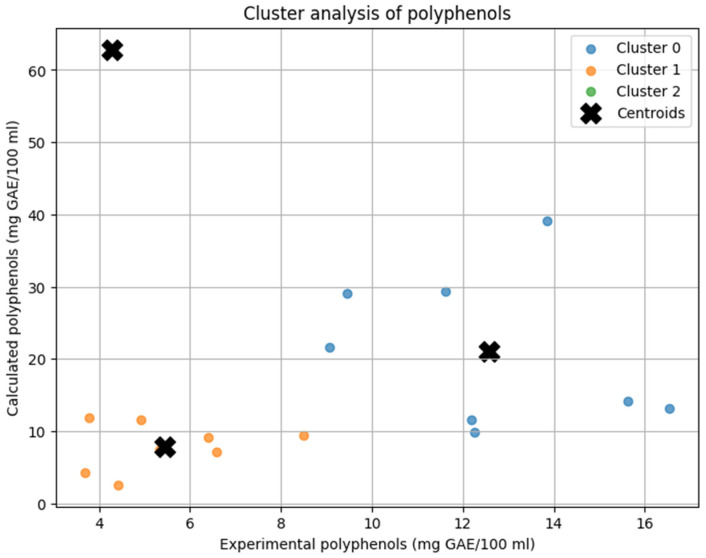
Cluster analysis of homemade soups based on polyphenol content (experimental vs. calculated).

The K-Means clustering analysis categorized soups into three distinct groups based on their experimental and calculated polyphenol content (mg GAE/100 mL) (Figure 1). Cluster 0 included soups with the highest polyphenol content, such as beetroot soup, broccoli soup, button mushroom cream soup, pumpkin cream soup, sour rye soup, tomato cream soup, tomato soup, and wild mushroom soup. In this group, theoretical calculations substantially overestimated the actual polyphenol levels. Cluster 1 comprised soups with lower polyphenol content, including barley soup, bean soup, button mushroom soup, chicken soup, cucumber soup, pea soup, sauerkraut soup, and vegetable soup. In these cases, experimental and calculated values showed better alignment, indicating a more accurate prediction by the theoretical model. Cluster 2 consisted of a single soup, green pea cream soup, which showed the largest discrepancy between measured and predicted values, with theoretical calculations extremely overestimating the actual polyphenol content.

The differences in clustering patterns reflected the accuracy of the predictive model. Cluster 1 demonstrated the best agreement between experimental and calculated values, suggesting that the model performs well for soups with lower polyphenol concentrations. Cluster 0 exhibited a noticeable tendency for overestimation, particularly for high-polyphenol soups. Cluster 2 consists of a single sample, which overlaps with its centroid on the plot. Its position—characterized by low experimental and high calculated polyphenol content—indicates a substantial overestimation by the predictive model for this sample.

### 3.2. Polyphenol Content in Ready-to-Eat Soups

The results for the polyphenol content in ready-to-eat soups, measured in mg GAE/100 mL, are presented in Table 2. The average polyphenol content in the ready-to-eat soups studied ranged from 4.387 mg GAE/100 mL in chicken soup to 18.531 mg GAE/100 mL in tomato soup. The tomato cream soup and beetroot soup were characterized by relatively high polyphenol content, amounting to 14.985 mg GAE/100 mL and 13.777 mg GAE/100 mL, respectively.

### 3.3. Polyphenol Content of Instant Soups

The polyphenol content results for instant soups are presented in mg GAE/100 mL (Table 3). Individual samples were available for tomato, cucumber, button mushroom, and broccoli soups. The highest average polyphenol content was found in tomato soup, with a mean of 7.254 mg GAE/100 mL, while the lowest was observed in white borscht samples, with 1.624 mg GAE/100 mL. Compared to tomato soup, the other soups had polyphenol levels that were several times lower. The greatest variability in results was found in beetroot soup, ranging from 1.312 to 7.717 mg GAE/100 mL.

### 3.4. Antioxidant Potential in Homemade Soups Measured and Calculated According to FRAP Method

The presented results of the antioxidant potential of homemade soups are expressed in mmol/100 g (Table 4). The highest average antioxidant activity was found in tomato cream soup (0.189 mmol/100 g), button mushroom soup (0.153 mmol/100 g), and wild mushroom soup (0.137 mmol/100 g). The lowest antioxidant potential was found for bean soup, chicken soup, barley soup, sauerkraut soup, and pea soup, at 0.21–0.28 mmol/100 g.

A comparison of the results obtained from experimental and calculation methods revealed significant variations, ranging from −62% in pumpkin cream soup to +524% in bean soup. The smallest discrepancies, not exceeding 10%, were observed in tomato soup and wild mushroom soup. The Sign test revealed a statistically significant predominance of overestimation in the calculated antioxidant potential (FRAP) values compared to experimental data (*p* = 0.025). This suggests a systematic bias in the predictive model.

**Figure 2:**
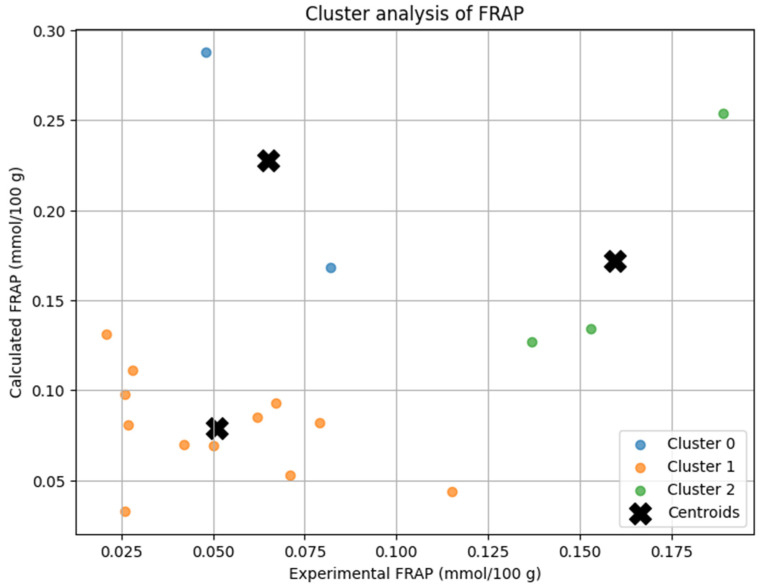
Cluster analysis of homemade soups based on FRAP antioxidant potential (experimental vs. calculated).

The K-Means clustering analysis grouped the soups into three distinct clusters based on their experimental and calculated FRAP antioxidant potential (Figure 2).

Cluster 0 included beetroot soup and broccoli soup, which exhibited higher antioxidant potential, where the calculated values were significantly overestimated compared to experimental results. Cluster 1 contained soups with lower antioxidant potential, including barley soup, bean soup, button mushroom soup, chicken soup, cucumber soup, green pea cream soup, pea soup, pumpkin cream soup, sauerkraut soup, sour rye soup, tomato soup, and vegetable soup, where the experimental and calculated values were relatively close, indicating better model agreement. Cluster 2 comprised button mushroom cream soup, tomato cream soup, and wild mushroom soup, which had moderate FRAP values, showing some overestimation but better agreement than Cluster 0.

The centroids of each cluster reflected the differences in antioxidant potential and model accuracy. Cluster 1 showed the highest agreement between experimental and calculated values, suggesting the model performs well for low-FRAP soups. Cluster 0 exhibited the largest deviation, indicating a tendency to overestimate antioxidant potential in high-FRAP samples. Cluster 2 had intermediate values, where theoretical predictions were still overestimated but closer to actual measurements.

### 3.5. Antioxidant Potential of Ready-to-Eat Soups by FRAP Method

The study assessed antioxidant activity in ready-to-eat soups using the FRAP method, with results expressed in mmol/100 g (Table 5). Beetroot soup exhibited the highest average antioxidant activity at 0.269 mmol/100 g, while chicken soup had the lowest at 0.029 mmol/100 g. Some soups, such as sour rye soup (0.063 mmol/100 g) and pumpkin cream soup (0.082 mmol/100 g), demonstrated higher antioxidant levels compared to others. Additionally, these soups showed relatively broad ranges between their minimum and maximum antioxidant values.

### 3.6. Antioxidant Potential by FRAP Method of Instant Soups

The FRAP antioxidant activity of instant soups is presented in Table 6. The average antioxidant content varies across different soup types, with tomato soup showing the highest level at 0.134 mmol/100 g, while white borscht had the lowest at 0.033 mmol/100 g.

### 3.7. Antioxidant Activity of Homemade Soups by Electrochemical Method

The results in Table 7 present the antioxidant activity in homemade soups, measured in microcoulombs (mC/g). The average antioxidant activity varied depending on the soup type, with broccoli soup showing the highest activity at 52.467 mC/g, followed closely by sauerkraut soup at 52.117 mC/g. In contrast, the lowest antioxidant activity was observed in button mushroom cream soup, with an average value of 22.410 mC/g.

### 3.8. Antioxidant Potential Measured Using Electrochemical Methods in Ready-to-Eat Soups

Table 8 presents the total antioxidant potential of ready-to-eat soups, measured in milicoulombs per gram (mC/g). The average antioxidant activity varies by soup type, with the highest levels found in pulse soups: green pea cream soup at 58.900 mC/g, bean soup at 57.480 mC/g, and pea soup at 54.973 mC/g. In contrast, chicken soup exhibited the lowest antioxidant potential, recorded at 22.750 mC/g.

### 3.9. Antioxidant Activity by Electrochemical Method in Instant Soups

Table 9 presents the antioxidant potential of various instant soups, expressed in milicoulombs per gram (mC/g). Button mushroom soup and broccoli soup exhibited the highest antioxidant potential, with mean values of 47.270 mC/g and 47.680 mC/g, respectively. Wild mushroom soup, white borscht, and sour rye soup showed lower antioxidant potential, with mean values of 32.310 mC/g, 27.115 mC/g, and 22.515 mC/g, respectively.

### 3.10. Correlations Between Experimental and Calculation Methods

**Figure 3:**
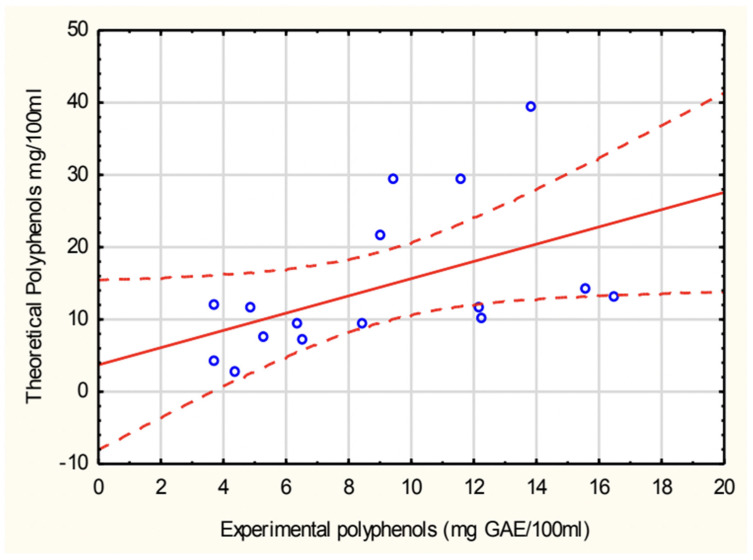
Correlation between experimental and calculated values of polyphenols in soups.

The correlation between experimentally determined and theoretically calculated polyphenol concentrations in soups is shown in Figure 3. A positive correlation was observed, with a Pearson correlation coefficient (r = 0.499), indicating a moderate relationship between the two datasets. The green pea cream soup values were excluded due to an extremely high calculated polyphenol concentration, which significantly deviated from experimental values.

**Figure 4:**
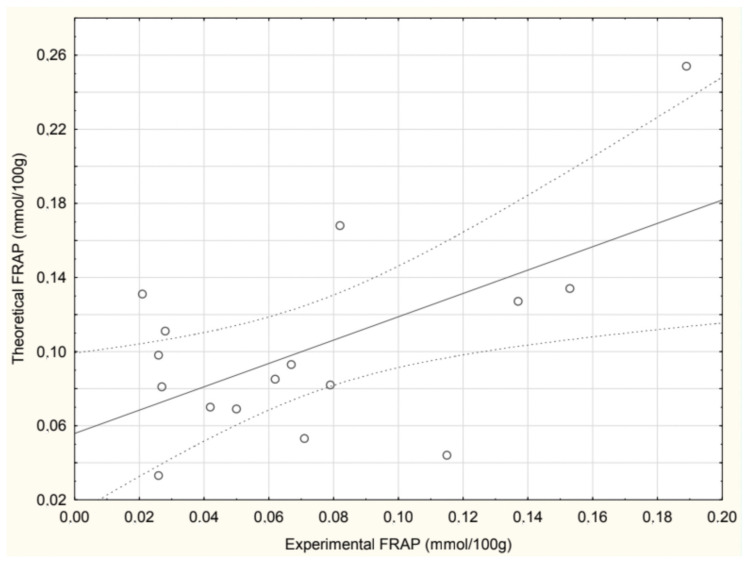
Correlation between experimental and calculated FRAP values in soups.

The correlation between the experimentally determined and theoretically calculated FRAP values in soups is presented in Figure 4. A positive correlation was observed, with a Pearson correlation coefficient r = 0.592, indicating a moderate but statistically significant relationship between the two datasets. The beetroot soup values were excluded from the analysis due to an extremely high calculated FRAP value, which significantly deviated from experimental measurements.

### 3.11. Correlations Between Polyphenols and Antioxidants Measured by FRAP and Electrochemical Method

**Figure 5:**
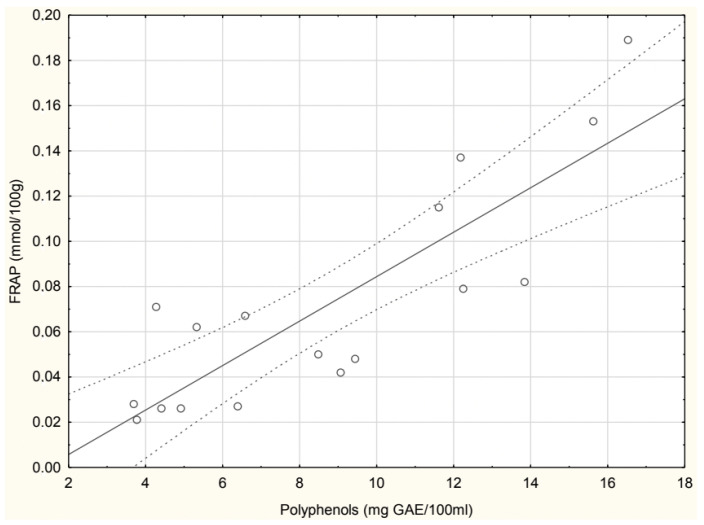
Correlation between experimental polyphenol and FRAP values in homemade soups.

A statistically significant positive correlation (r = 0.848) was observed between polyphenol content (mg GAE/100 mL) and FRAP values (mmol/100 g), as shown in Figure 5. The regression analysis indicates that higher polyphenol concentrations correspond to increased antioxidant capacity, confirming the strong relationship between these parameters.

**Figure 6:**
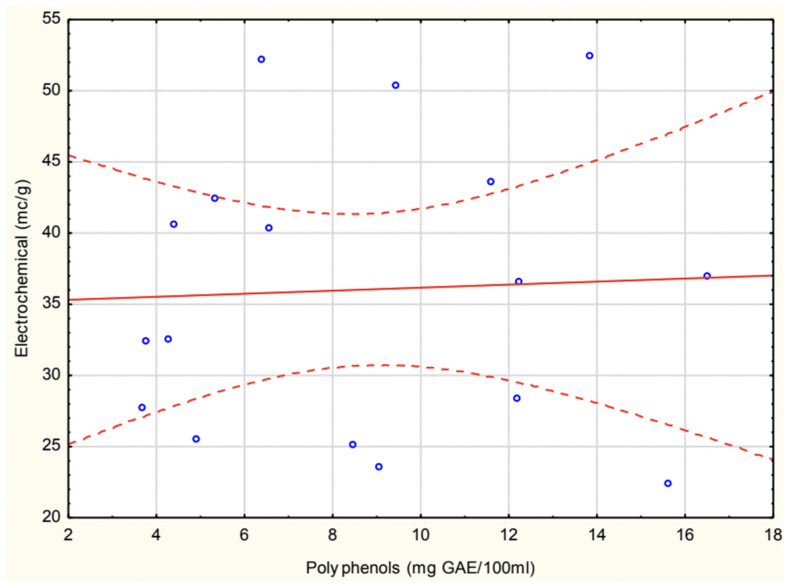
Correlation between experimental polyphenol and electrochemical methods in homemade soups.

The correlation analysis between polyphenol content and electrochemical antioxidant activity (Figure 6) showed an insignificant relationship (r = 0.046), indicating no meaningful linear association.

**Figure 7:**
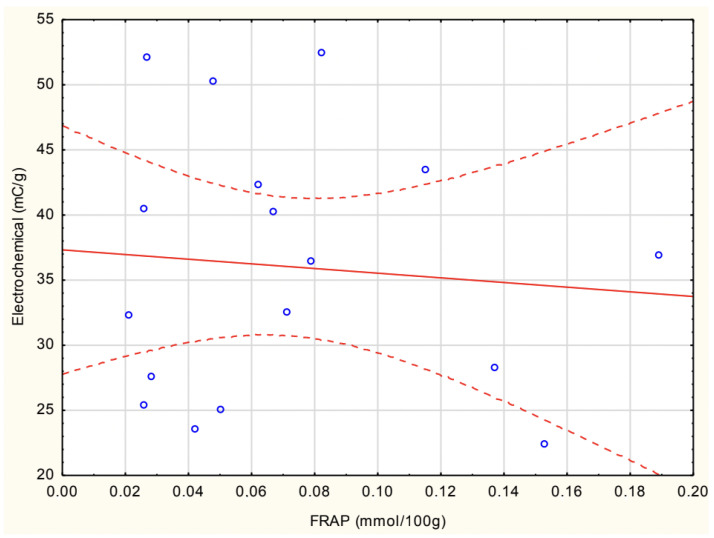
Correlation between experimental FRAP and electrochemical methods in homemade soups.

Figure 7 shows the correlation between experimental FRAP and electrochemical antioxidant activity in homemade soups. The correlation found was insignificant (r = −0.089).

### 3.12. Comparisons Between Polyphenol Content, FRAP Antioxidant Capacity, and Electrochemical Antioxidant Activity for Selected Homemade, Ready-to-Eat, and Instant Soups

The selection of soups for analysis was based on the availability of all three processing categories: homemade, ready-to-eat, and instant. Only soups that were available in all three forms (beetroot soup, cucumber soup, pea soup, tomato soup) were included to ensure a consistent basis for comparison.

**Figure 8:**
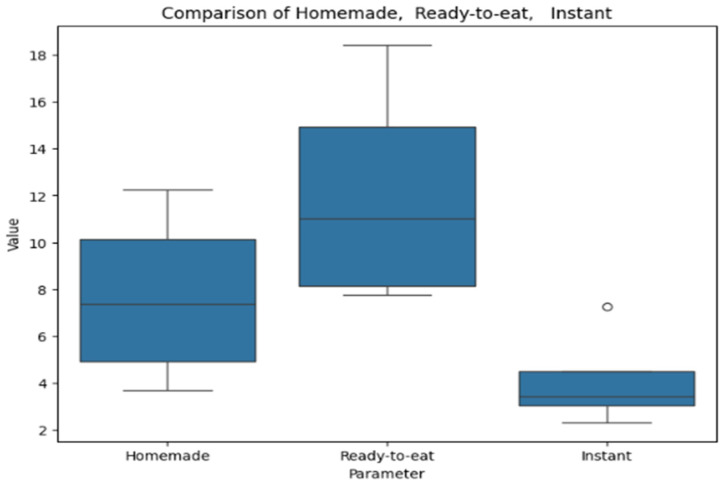
Box plot comparing polyphenol levels in homemade, ready-to-eat, and instant categories.

Figure 8 provides a box plot representing polyphenol content in soups. The box plot compares values across homemade, ready-to-eat, and instant soups. Ready-to-eat soups exhibit the highest median and the widest range, indicating greater variability. Homemade soups have a lower median with moderate dispersion. Instant soups show the lowest values, with minimal variation except for a single outlier.

**Figure 9:**
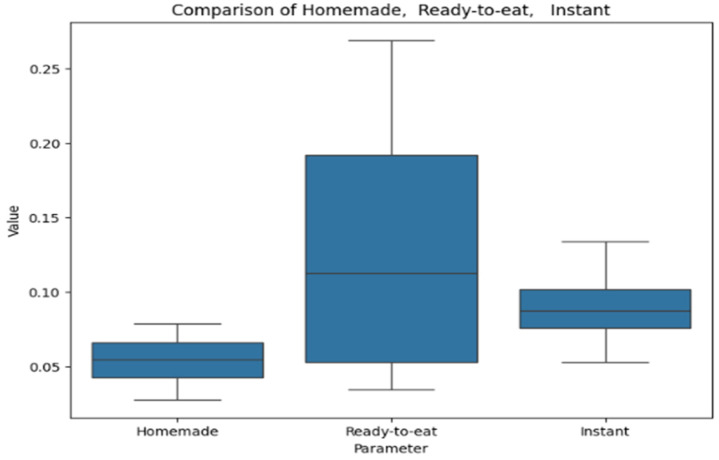
Box plot comparing antioxidant activity by FRAP in homemade, ready-to-eat, and instant categories.

Figure 9 presents the results of the FRAP assay for soups, visualized as a box plot. The box plot compares values across homemade, ready-to-eat, and instant soups. Homemade soups show the lowest values with minimal variation, while ready-to-eat soups exhibit the highest variability and a wider range. Instant soups have intermediate values with moderate dispersion.

**Figure 10:**
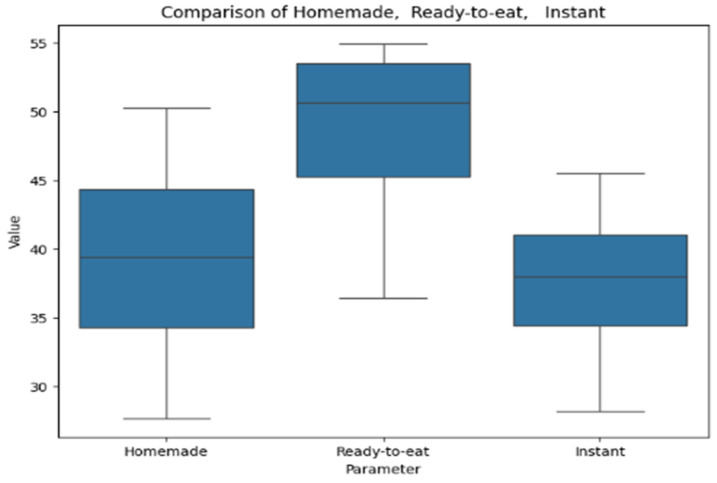
Box plot comparing antioxidant activity by electrochemical method in homemade, ready-to-eat, and instant categories.

Figure 10 provides a box plot representing the analysis of soups by the electrochemical method for antioxidant activity. The box plot compares values across homemade, ready-to-eat, and instant soups. Ready-to-eat soups show the highest median and variability, homemade soups have a lower median with a broader range, and instant soups exhibit the lowest median and the most consistent values.

## 4. Discussion

This study presents an analysis of polyphenol content by the spectrophotometric method of Singleton and Rossi [18] and antioxidant activity using two distinct methods (FRAP and electrochemical) in various Polish traditional soups, prepared through different approaches: homemade soups, ready-to-eat soups, and instant soups. Unlike previous research, which typically focused on examining polyphenol content or antioxidant activity in a single type of dish or comparing raw ingredients with their processed counterparts, this study offers a comparison across multiple preparation methods [21,22,23,24,25]. This study represents the first effort to assess polyphenol content and FRAP values using both experimental and calculated data, allowing for a comparison of results between these two methods in model dishes. In this study, the model dishes were soups, which had the advantage of preventing losses associated with leaching and removal of nutrients and biologically active compounds when draining the broth, as these substances remain in the water in which the food was cooked.

Although experimental and calculated values showed a correlation for both polyphenols and FRAP in homemade soups, the results suggest that there may be differences in polyphenol and FRAP values between the two methods, potentially attributable to various factors. The clustering analysis revealed systematic overestimation of polyphenol content and FRAP antioxidant potential in theoretical calculations, particularly in high-polyphenol and high-antioxidant soups such as beetroot soup and broccoli soup. Statistical analysis indicated a consistent overestimation of both polyphenol content and antioxidant potential, with a significant bias confirmed for FRAP values (*p* = 0.025), suggesting limitations in the predictive accuracy of food composition databases for homemade soups. In contrast, soups with lower polyphenol and FRAP values, including, for example, bean soup and chicken soup, showed better agreement between experimental and calculated values, indicating greater model accuracy for these products. A notable outlier was green pea cream soup, where calculated polyphenol content was overestimated, suggesting theoretical models do not fully capture compound stability in certain matrices. Similarly, the FRAP clustering revealed that theoretical values were inflated for high-antioxidant soups, likely due to processing losses not being adequately accounted for in predictive models. These findings highlight the limitations of current theoretical models, which do not fully consider processing effects, degradation kinetics, and ingredient interactions. Therefore, it may be necessary to review the tabulated results for the validity of the research sources from which they were compiled, especially given that the databases predominantly provide values for raw products and frequently lack corresponding values for items subjected to culinary processing. Furthermore, the research methodologies themselves may lack sufficient accuracy, as they do not comprehensively analyze all antioxidants present in the sample, including polyphenolic compounds and other non-phenolic antioxidants, and therefore each of them has its limitations [26]. Additionally, the thermal processing of food can affect the increase or decrease in polyphenol content and antioxidant potential [27]. On one hand, it can facilitate the softening of products, thereby promoting the easier extraction of biologically active components; on the other hand, it may lead to the degradation of certain important antioxidant substances [11].

The Folin method has long been used to measure the total polyphenol content in food, while the FRAP method has been employed to assess antioxidant potential, and based on these studies, relevant databases have been developed [15,16]. This study provides antioxidant potential values determined using the electrochemical method, which is more versatile and cost-effective than chemical methods [28]. Unlike the FRAP method, which requires the use of reagents, the electrochemical method enables direct measurement on the device’s electrode. The absence of chemicals reduces negative environmental impact, allowing for faster and more efficient measurements, and contributes to environmental sustainability [29]. This study provides the first dataset assessing antioxidant activity in culinary dishes using this method.

In all methods of soup preparation, tomato soups consistently showed the highest polyphenol content. Tomatoes are a rich source of phenolic compounds, such as chlorogenic acid, caffeic acid, and naringenin, all of which possess strong antioxidant properties as well as carotenoid lycopene [30]. Generally, thermal processing improves the bioavailability of these compounds, contributing to the higher polyphenol content found in tomato-based products [31,32,33].

Among the soups assayed, beetroot soup, in homemade or ready-to-eat forms, demonstrated high polyphenol content. Bioactive compounds naturally found in beets include betalains, ferulic acid, caffeic acid, and other polyphenols. Betalains, particularly betanin and vulgaxanthin, are powerful antioxidants, and their concentrations remain relatively stable even after thermal processing [34,35].

Generally, instant soups exhibited lower polyphenol content and antioxidant activity by the electrochemical method compared to homemade and ready-to-eat soups. Intensive industrial processes, including drying and heat treatment, may contribute to the degradation of polyphenols, which are sensitive to high temperatures, oxygen, and light, leading to their breakdown during production [36,37]. Furthermore, instant soups typically contain fewer fresh ingredients, such as vegetables, which are natural sources of polyphenols, further reducing the polyphenol content in the final product. The lower polyphenol content may also result from different proportions of bioactive ingredients in instant soups, which, in addition to dehydrated vegetables and meat, may contain flavor enhancers, starch, fats, and preservatives [38].

The correlation analysis revealed varying degrees of association between polyphenol content, FRAP antioxidant potential, and electrochemical antioxidant activity. The correlation between experimental and calculated polyphenol content was moderate, indicating that while theoretical models provide an estimation, they do not fully capture the effects of processing on polyphenol retention. The relationship between FRAP and polyphenols was stronger, suggesting that polyphenol content contributes significantly to FRAP-based antioxidant capacity. However, the correlation between FRAP and electrochemical activity was weak and insignificant, indicating that these two antioxidant assessment methods likely capture different aspects of antioxidant potential. While FRAP primarily measures ferric ion-reducing potential [19], the electrochemical method detects the total redox activity, which may be influenced by additional factors such as oxidation state changes, interactions with other food components, or the presence of non-polyphenolic antioxidants. The observed discrepancies highlight the complexity of antioxidant behavior in processed foods and the limitations of relying on a single analytical method.

The variability in correlations also underscores the importance of direct experimental measurements over theoretical estimations, as processing effects on polyphenol stability and antioxidant activity are not fully predictable using calculation models alone.

## 5. Limitations

This study presents several limitations. First, the availability of commercial soup products varied significantly across categories. For some soup types, only a single product variant was available, resulting in single-sample measurements. While all homemade soups were analyzed in triplicate, this level of replication could not be achieved for all ready-to-eat and instant products due to market limitations.

Second, the detailed quantitative composition of commercial soups could not be assessed due to food labeling regulations in Poland. Manufacturers are not required to declare the exact proportion of individual ingredients, which limited our ability to perform ingredient-level comparisons between products. Therefore, only general composition information was available based on the declared ingredient lists.

To enhance transparency, Supplementary Table S2 provides the product names, manufacturers, and formats (ready-to-eat or instant), allowing for independent identification and evaluation of the products used. These limitations should be taken into account when interpreting the variability and representativeness of antioxidant data in commercial soups.

## 6. Conclusions

This study demonstrates discrepancies between experimentally determined and calculated values for total polyphenol content and antioxidant activity in soups, with a statistically significant overestimation observed for antioxidant activity. These findings suggest that theoretical estimations may not fully account for the effects of processing and ingredient interactions. This is particularly relevant for dietary recommendations and nutritional assessments, where overestimation of polyphenol and antioxidant content may lead to misinterpretations of food quality and associated health benefits.

These findings emphasize the need for direct experimental analysis to more accurately assess the nutritional properties of processed foods and to ensure accurate inclusion in food composition databases.

## Figures and Tables

**Table 1:** Polyphenol content of homemade soups obtained by experimental and calculation methods.

Polyphenol Content in mg GAE/100 mL								
Experimental							Calculated	% Difference for Calculation Method
	n	Mean	SD	Median	Min	Max		
Barley soup	3	4.919	0.947	4.766	4.058	5.933	11.525	+134
Bean soup	3	3.778	0.966	3.265	3.177	4.893	11.825	+213
Beetroot soup	3	9.444	0.008	9.444	9.438	9.450	29.148	+209
Broccoli soup	3	13.843	0.953	13.798	12.914	14.818	39.129	+183
Button mushroom cream soup	3	15.628	0.251	15.553	15.424	15.908	14.130	−10
Button mushroom soup	3	6.583	0.535	6.379	6.180	7.190	7.192	+8
Chicken soup	3	4.415	1.033	4.151	3.540	5.555	2.582	−42
Cucumber soup	3	5.330	0.326	5.455	4.960	5.574	7.391	+39
Green pea cream soup	3	4.275	0.337	4.206	3.978	4.641	62.827	+1370
Pea soup	3	3.692	0.370	3.865	3.268	3.944	4.205	+14
Pumpkin cream soup	3	11.618	1.611	11.286	10.198	13.369	29.432	+153
Sauerkraut soup	3	6.393	0.369	6.453	5.998	6.728	9.184	+44
Sour rye soup	3	9.069	0.986	9.218	8.017	9.973	21.585	+138
Tomato cream soup	3	16.534	1.112	16.742	15.332	17.527	13.137	−21
Tomato soup	3	12.255	1.525	11.839	10.981	13.945	9.917	−19
Vegetable soup	3	8.489	0.4793	8.758	7.936	8.774	9.397	+11
Wild mushroom soup	3	12.186	0.471	12.207	11.705	12.646	11.564	−5

SD—standard deviation.

**Table 2:** Polyphenol content of ready-to-eat soups by experimental method.

Polyphenol Content in mg GAE/100 mL						
	n	Mean	SD	Median	Min	Max
Barley soup	3	5.604	2.245	4.580	4.054	8.179
Bean soup	1	8.896 *	-	-	-	-
Beetroot soup	2	13.777	1.018	13.777	13.057	14.496
Button mushroom cream soup	1	8.362 *	-	-	-	-
Chicken soup	1	4.387 *	-	-	-	-
Cucumber soup	4	7.750	0.693	7.722	6.935	8.619
Pea cream soup	1	6.505 *	-	-	-	-
Pea soup	4	8.248	0.914	8.183	7.392	9.233
Pumpkin cream soup	1	11.819 *	-	-	-	-
Sauerkraut soup	3	9.991	2.772	10.241	7.102	12.630
Sour rye soup	3	10.604	3.575	10.331	7.174	14.306
Tomato cream soup	1	14.985 *	-	-	-	-
Tomato soup	3	18.431	1.572	18.431	17.319	19.542
Vegetable soup	3	6.865	0.674	7.223	6.087	7.285

SD—standard deviation; * single value.

**Table 3:** Polyphenol of content of instant soups by experimental method.

Polyphenol Content in mg GAE/100 mL						
	n	Mean	SD	Median	Min	Max
Beetroot soup	3	3.583	3.585	1.722	1.312	7.717
Broccoli soup	1	3.228 *	-	-	-	-
Button mushroom soup	1	2.081 *	-	-	-	-
Cucumber soup	1	2.322 *	-	-	-	-
Pea soup	2	3.267 *	1.609	3.267	2.129	4.405
Sour rye soup	2	3.585	0.977	3.585	2.894	4.276
Tomato soup	1	7.254 *	-	-	-	-
White borscht	2	1.624	0.624	1.624	1.183	2.065
Wild mushroom soup	2	1.712	0.827	1.712	1.127	2.296

SD—standard deviation; * single value.

**Table 4:** FRAP antioxidant potential of homemade soups by experimental and calculation methods.

Antioxidant Potential in mmol/100 g								
Experimental							Calculated	% Difference for Calculation Method
	n	Mean	SD	Median	Min	Max		
Barley soup	3	0.026	0.002	0.025	0.024	0.028	0.098	+277
Bean soup	3	0.021	0.004	0.019	0.018	0.026	0.131	+524
Beetroot soup	2	0.048	0.029	0.048	0.027	0.068	0.288	+500
Broccoli soup	3	0.082	0.004	0.080	0.079	0.087	0.168	+105
Button mushroom cream soup	3	0.153	0.006	0.150	0.149	0.159	0.134	−12
Button mushroom soup	3	0.067	0.011	0.067	0.056	0.078	0.093	+39
Chicken soup	3	0.026	0.060	0.028	0.020	0.031	0.033	+27
Cucumber soup	3	0.062	0.003	0.062	0.059	0.066	0.085	+37
Green pea cream soup	3	0.071	0.006	0.071	0.065	0.076	0.053	−25
Pea soup	3	0.028	0.003	0.029	0.024	0.030	0.111	+296
Pumpkin cream soup	3	0.115	0.015	0.118	0.098	0.128	0.044	−62
Sauerkraut soup	3	0.027	0.006	0.028	0.021	0.032	0.081	+200
Sour rye soup	3	0.042	0.005	0.043	0.036	0.046	0.070	+67
Tomato cream soup	3	0.189	0.012	0.188	0.178	0.202	0.254	+34
Tomato soup	3	0.079	0.011	0.080	0.068	0.090	0.082	+4
Vegetable soup	3	0.050	0.011	0.044	0.043	0.063	0.069	+38
Wild mushroom soup	3	0.137	0.013	0.134	0.127	0.152	0.127	−7

SD—standard deviation.

**Table 5:** Antioxidant potential measured by the FRAP method in ready-to-eat soups.

Antioxidant Potential in mmol/100 g						
	n	Mean	SD	Median	Min	Max
Barley soup	3	0.038	0.010	0.036	0.030	0.049
Bean soup	1	0.049 *	-	-	-	-
Beetroot soup	2	0.269	0.087	0.269	0.207	0.330
Cabbage soup	3	0.053	0.019	0.059	0.032	0.068
Chicken soup	1	0.029 *	-	-	-	-
Cucumber soup	4	0.035	0.004	0.036	0.030	0.038
Mushroom cream soup	1	0.057 *	-	-	-	-
Pea cream soup	1	0.059 *	-	-	-	-
Pea soup	4	0.036	0.006	0.037	0.028	0.042
Pumpkin cream soup	1	0.082 *	-	-	-	-
Sour rye soup	3	0.063	0.038	0.060	0.027	0.102
Tomato cream soup	1	0.139 *	-	-	-	-
Tomato soup	2	0.166	0.048	0.166	0.132	0.200
Vegetable soup	3	0.031	0.022	0.026	0.012	0.056

SD—standard deviation; * single value.

**Table 6:** Antioxidant potential measured by the FRAP method in instant soups.

Antioxidant Potential in mmol/100 g						
	n	Mean	SD	Median	Min	Max
Beetroot soup	3	0.084	0.095	0.036	0.022	0.193
Broccoli soup	1	0.055 *	-	-	-	-
Button mushroom soup	1	0.058 *	-	-	-	-
Cucumber soup	1	0.091 *	-	-	-	-
Pea soup	2	0.053	0.015	0.053	0.042	0.063
Sour rye soup	2	0.078	0.018	0.078	0.065	0.090
Tomato soup	1	0.134 *	-	-	-	-
White borscht	2	0.033	0.012	0.033	0.024	0.041
Wild mushroom soup	2	0.067	0.060	0.067	0.024	0.109

SD—standard deviation; * single value.

**Table 7:** Antioxidant activity by electrochemical method of homemade soups.

Antioxidant Potential (mC/g)						
	n	Mean	SD	Median	Min	Max
Barley soup	3	25.480	6.626	28.020	17.960	30.460
Bean soup	3	32.350	1.443	32.350	31.330	33.370
Beetroot soup	2	50.297	1.581	50.230	48.750	51.910
Broccoli soup	3	52.467	5.087	53.720	46.870	56.810
Button mushroom cream soup	3	22.410	3.208	20.720	20.400	26.110
Button mushroom soup	3	40.297	6.452	43.830	32.850	44.210
Chicken soup	3	40.527	1.382	40.620	39.100	41.860
Cucumber soup	3	42.367	3.969	44.310	37.800	44.990
Pea cream soup	3	32.553	0.973	32.170	31.830	33.660
Pea soup	3	27.650	4.102	28.330	23.250	31.370
Pumpkin cream soup	3	43.550	12.028	41.970	32.390	56.290
Sauerkraut soup	3	52.117	14.847	60.020	34.990	61.340
Sour rye soup	3	23.593	3.996	22.760	20.080	27.940
Tomato cream soup	3	36.967	4.995	37.030	31.940	41.930
Tomato soup	3	36.493	3.156	38.240	32.850	38.390
Vegetable soup	3	25.110	3.813	24.250	21.800	29.280
Wild mushroom soup	3	28.317	3.522	30.310	24.250	30.390

SD—standard deviation.

**Table 8:** Antioxidant activity of ready-to-eat soups measured with electrochemical method.

Antioxidant Potential (mC/g)						
	n	Mean	SD	Median	Min	Max
Barley soup	3	39.067	2.869	37.630	37.200	42.370
Bean soup	1	57.480 *	-	-	-	-
Beetroot soup	2	48.240	3.437	48.240	45.810	50.670
Button mushroom cream soup	1	39.230 *	-	-	-	-
Chicken soup	1	22.750 *	-	-	-	-
Cucumber soup	4	36.413	2.014	36.550	34.140	38.410
Green pea cream soup	1	58.900 *	-	-	-	-
Pea soup	4	54.973	5.292	55.115	48.860	60.800
Pumpkin cream soup	1	41.490 *	-	-	-	-
Sauerkraut soup	3	44.643	10.156	41.300	36.580	56.050
Sour rye soup	3	50.270	17.438	58.690	30.220	61.900
Tomato cream soup	1	53.040 *	-	-	-	-
Tomato soup	3	53.080	12.629	53.080	44.150	62.010
Vegetable soup	3	36.087	7.584	38.890	27.500	41.870

SD—standard deviation; * single value.

**Table 9:** Antioxidant activity of instant soups measured with electrochemical method.

Antioxidant Potential (mC/g)						
	n	Mean	SD	Median	Min	Max
Beetroot soup	3	28.177	8.389	24.670	22.110	37.750
Broccoli soup	1	47.680 *	-	-	-	-
Button mushroom soup	1	47.270 *	-	-	-	-
Cucumber soup	1	45.510 *	-	-	-	-
Pea soup	2	39.565	7.36	39.565	34.590	44.540
Sour rye soup	2	22.515	2.246	22.515	20.930	24.100
Tomato soup	1	36.450 *	-	-	-	-
White borscht	2	27.115	4.419	27.115	23.990	30.240
Wild mushroom soup	2	32.310	3.748	32.310	29.660	34.960

SD—standard deviation; * single value.

## Data Availability

The original contributions presented in this study are included in the article and Supplementary Materials. Further inquiries can be directed to the corresponding author.

## Supplementary Materials

The following supporting information can be downloaded at: https://www.mdpi.com/article/10.3390/antiox14050563/s1.
