# Supplementary material for: Polyphenol Content and Antioxidant Activity in Homemade and Commercial Soups: A Theoretical and Experimental Approach
Source: Antioxidants (Basel). 2025 May 8;14(5):563. doi: 10.3390/antiox14050563 (PMC12108172; doi:10.3390/antiox14050563)
Supplement: Supplementary file 1 [file antioxidants-14-00563-s001.zip › Supplementary Table S1.pdf]

Supplementary Table S1.

Soup/Ingredient	Percentage (per 100 g)
**Vegetable-meat broth**	
pork bones (loin and neck)	9.2
leek	1.68
parsley root	1.47
carrot	7.16
celeriac	2.27
water	78.22
total	100
**Vegetable soup**	
vegetable-meat broth	47.58
green beans	19.08
carrot	9.5
parsley root	2.42
leek	2.42
celeriac	2.42
potatoes	14.25
soft margarine, 80% fat	1.17
parsley leaves	1.17
total	100
**Cucumber soup**	
vegetable-meat broth	67
pickled cucumber	9.19
potatoes	25.64
soft margarine, 80% fat	1.29
sour cream, 18% fat	5.51
wheat flour	1.10
sugar	0.28

total	100
**Tomato soup**	
vegetable-meat broth	73.49
carrot	9.14
parsley root	3.73
leek	3.73
celeriac	1.8
tomato paste 30%	5.53
sugar	0.77
parsley leaves	1.8
total	100
**Button mushroom soup**	
vegetable-meat broth	80.32
button mushroom	8.84
onion	2
butter	1.2
sour cream, 18% fat	6.02
wheat flour	1.2
parsley leaves	0.4
total	100
**Wild mushroom soup**	
vegetable-meat broth	80.32
wild mushrooms	8.84
onion	2
butter	1.2
sour cream, 18% fat	6.02
wheat flour	1.2
parsley leaves	0.4
total	100

**Pea soup**	
vegetable-meat broth	74.36
yellow peas	20.41
pork bacon	3.82
lard	1.28
wheat flour	0.51
total	100
**Sauerkraut soup**	
vegetable-meat broth	79.6
sauerkraut	13.72
button mushroom	1
onion	0.91
pork bacon	2.2
wheat flour	0.91
sugar	0.37
parsley leaves	0.37
total	100
**Sour rye soup**	
sour rye soup concentrate	63.49
carrot	5.12
potatoes	20.13
sour cream, 18% fat	4.33
egg	3.61
lard	1.15
onion	2.16
total	100
**Barley soup**	
vegetable-meat broth	53.31
rice	4.01

carrot	6.63
parsley root	2.71
leek	2.71
celeriac	1.3
potatoes	26.72
soft margarine, 80% fat	1.31
parsley leaves	1.31
total	100
**Bean soup**	
vegetable-meat broth	72.78
white beans	22.91
lard	2.7
wheat flour	1.08
parsley leaves	0.54
total	100
**Beetroot soup**	
vegetable-meat broth	67.8
beetroot	19.26
onion	2.31
button mushroom	0.85
sour cream, 18% fat	4.62
wheat flour	0.77
sugar	0.54
egg	3.85
total	100
**Green pea cream soup**	
vegetable-meat broth	50.06
green peas	39

butter	1.92
sour cream, 18% fat	7.85
wheat flour	0.8
sugar	0.32
parsley leaves	0.32
total	100
**Button mushroom cream soup**	
vegetable-meat broth	50.06
button mushroom	39
butter	1.92
sour cream, 18% fat	7.85
wheat flour	0.8
sugar	0.32
parsley leaves	0.32
total	100
**Broccoli soup**	
vegetable-meat broth	47.58
broccoli	19.08
carrot	9.5
parsley root	2.42
leek	2.42
celeriac	2.42
potatoes	14.25
soft margarine, 80% fat	1.17
parsley leaves	1.17
total	100
**Pumpkin cream soup**	
vegetable-meat broth	50.06

pumpkin	39
butter	1.92
sour cream, 18% fat	1.92
wheat flour	0.8
sugar	0.32
parsley leaves	0.32
total	100
**Tomato cream soup**	
vegetable-meat broth	50.06
canned tomatoes	39
butter	1.92
sour cream, 18% fat	1.92
wheat flour	0.8
sugar	0.32
parsley leaves	0.32
total	100
**Chicken soup**	
vegetable-meat broth	77.70
vegetables	9.57
chicken meat	7.66
chicken fat	0.77
rice	4.30
total	100
