# Supplementary material for: Polyphenol Content and Antioxidant Activity in Homemade and Commercial Soups: A Theoretical and Experimental Approach
Source: Antioxidants (Basel). 2025 May 8;14(5):563. doi: 10.3390/antiox14050563 (PMC12108172; doi:10.3390/antiox14050563)
Supplement: Supplementary file 1 [file antioxidants-14-00563-s001.zip › Supplementary Table S2.pdf]

Supplementary Table S2.

Ready-to-eat soups		
Soup Type	Ingredients	Manufacturer
Barley soup	Water, vegetables (potatoes 5%, carrots 4%, celery), pork meat (6%), barley groats (2.6%), cream (milk), modified starch, wheat flour, salt, dill, black pepper, yeast extract, antioxidant: sodium ascorbate.	P
Barley soup	Water, barley groats (11.5%), potatoes (5%), carrot, celery, chicken broth (chicken extract, glucose, yeast extract, flavorings, maltodextrin, turmeric, barley malt extract), dried vegetables (carrot, celery, onion, garlic, parsley), wheat flour, thickener: modified corn starch, salt, spices.	K
Barley soup	Water, potatoes, chicken meat pieces (7%) (chicken meat, pea fiber, chicken proteins, salt), onion, barley groats (3%), carrot, wheat flour, spice extracts, salt, glucose, spices (contain celery), celery, dill, dried vegetables (parsnip, carrot, onion, parsley leaves).	J
Bean soup	Spring water, white beans "Jaś" (22%), pasta (9%) (gluten), pork meat (4.5%), pork sausage (4.5%) (pork meat, water, salt, spices [mustard], sugar), onion (4%), carrot (4%), tomato paste, rapeseed oil, vegetable broth [vegetable broth base (69.6%) (water, salt, yeast extract, vegetables (carrot, onion, leek, garlic, celery root), sunflower oil, corn starch, concentrated lemon juice, sea salt), water, sugar, corn starch, flavourings, turmeric], salt, garlic, dried vegetables, red paprika powder, lard, natural and flavour-enhancing spices, marjoram, black pepper.	D
Beetroot soup	Beetroot juice from concentrated juice (58%), water, apple juice from concentrated juice, sugar, salt, acidity regulator: citric acid, vegetables and vegetable extracts (contain celery), spices and spice extracts, flavourings (contain celery, milk).	K
Beetroot soup	Juices from concentrated juices: beetroot (60%), apple (22%); vegetable juice (3.8%) in variable proportions from: carrot, celery, parsley root, onion; salt, spice extract (black pepper, allspice, bay leaf).	H
Button mushroom cream soup	Water, mushrooms (18%), potatoes, cream (milk), onion, celery, modified starch, parsley, carrot, salt, black pepper, yeast extract, antioxidant: sodium ascorbate.	J
Chicken soup	Water, vegetables (10%) (carrot, celery, onion), chicken meat (8%), rice (4.5%), modified starch, salt,	J

	chicken fat (0.8%), black pepper, lovage, yeast extract, antioxidant: sodium ascorbate.	
Cucumber soup	Water, pickled cucumbers (22%) (cucumbers, salt, garlic, dill), potatoes, carrots, onion, turkey meat, parsley root, celery, cream (milk), rapeseed oil, dill (0.3%), salt, spices.	B
Cucumber soup	Pickled cucumbers (40%) (cucumbers, salt, spices [including mustard seeds]), potatoes, carrots, parsley root, celery root, dill (3%), cream (milk), water, salt, sugar.	S
Cucumber soup	Water, pickled cucumbers (21%) (cucumbers, salt), vegetables (potatoes 6.5%, carrots 3%, celery), pork meat (4%), cream (milk), wheat flour, modified starch, salt, dill, black pepper, yeast extract, antioxidant: sodium ascorbate.	P
Cucumber soup	Chicken broth (water, chicken carcass, salt), grated pickled cucumbers (17%) (cucumbers, water, salt, spices), vegetables in variable proportions (carrot, potato, celery, parsley root), wheat flour, cream (from milk) (1%), salt, dill (0.2%), sugar, garlic (0.1%), spices, spice extracts.	L
Pea cream soup	Water, green peas (30%), potatoes, onion, cream (milk), rapeseed oil, salt, black pepper, paprika, yeast extract, antioxidant: sodium ascorbate.	J
Pea soup	Yellow peas (32%), spring water, potatoes (12%), onion (5%), pork sausage (3%) (pork meat, water, salt, spices [mustard, sugar]), pork bacon (3%) (pork meat, water, salt, black pepper), garlic, salt, vegetable broth [vegetable broth base (69.6%) (water, salt, yeast extract, vegetables [carrot, onion, leek, garlic, celery root], sunflower oil, corn starch, concentrated lemon juice, sea salt), water, sugar, corn starch, flavorings, turmeric], natural spices and flavorings (dried vegetables, marjoram, black pepper, bay leaf, parsley leaves).	D
Pea soup	Water, split peas (26%), smoked pork sausage (pork meat, water, salt, spices [including mustard], modified starch, sugar), potatoes, carrots, onion, smoked pork fat, wheat flour, modified corn starch, garlic, salt, black pepper, marjoram, yeast extract.	J
Pea soup	Water, vegetables (10%) (potatoes, carrots, celery), peas (8%), pork sausage (6%) (pork meat, salt, modified starch, spices, sugar), smoked pork fat, modified starch, wheat flour, salt, black pepper, marjoram, onion, yeast extract, antioxidant: sodium ascorbate.	P

Pea soup	Water, yellow split peas (24%), potatoes, smoked bacon (pork meat, water, salt, antioxidant: sodium ascorbate), carrots, onions, pork sausage (pork meat, water, salt, spices [including mustard], sugar), modified corn starch, wheat flour, salt, garlic, black pepper, marjoram, yeast extract.	L
Pumpkin cream soup	Water, pumpkin (30%), potatoes, onion, carrot, cream (milk), rapeseed oil, butter (milk), modified starch, salt, spices, sugar, yeast extract, antioxidant: sodium ascorbate.	J
Sauerkraut soup	Fresh white cabbage, sauerkraut (30%), spring water, potatoes (20%), tomato paste (4%), onion (4%), carrot (3%), vegetable oil (rapeseed or sunflower), natural spices (bay leaf, allspice, marjoram).	D
Sauerkraut soup	Water, sauerkraut (20%) (white cabbage, salt), vegetables (9%) (carrots, potatoes, celery), pork meat (5%), wheat flour, modified starch, smoked pork fat, salt, black pepper, spices (including: black pepper, cumin), yeast extract, antioxidant: sodium ascorbate.	P
Sauerkraut soup	Water, sauerkraut (22%) (white cabbage, salt), vegetables (potatoes, onion, carrot 2%), pork and beef sausage (3.5%) (pork meat, beef meat, salt, spices, soy protein isolate, stabilizers: diphosphates, triphosphates; potato starch, flavour enhancer: monosodium glutamate, preservative: sodium nitrite), smoked bacon (3%) (pork meat, water, salt, stabilizers: diphosphates, triphosphates; soy protein, antioxidant: sodium isoascorbate, preservative: sodium nitrite, spices), wheat flour, tomato paste, salt, sugar, pork fat, spices, flavourings.	A
Sour rye soup	Water, sourdough (20%) (water, rye flour, garlic), pork sausage (10%) (pork meat, salt, modified starch, spices, sugar), cream (milk) (2%), modified starch, wheat flour, salt, smoked pork fat, roasted onion (0.4%) (onion, palm oil, wheat flour, salt), garlic, rapeseed oil, marjoram, black pepper, yeast extract, antioxidant: sodium ascorbate.	P
Sour rye soup	Spring water, pork sausage (8%) (pork meat, water, salt, spices [mustard], sugar), flour (wheat, rye, oat), fresh garlic (0.2%), natural and flavor-enhancing spices (salt, allspice, bay leaf, ground black pepper, marjoram).	D
Sour rye soup	Water, vegetable purées in variable proportions (4%) (from onion, potatoes), flours (rye and wheat), natural rye sourdough (1%), pork fat, salt, skimmed milk powder, lactose, milk protein, mushroom extract, spices and spice extracts (including garlic), marjoram (0.05%), acidity regulators (lactic acid, citric acid), flavourings	K

	(contain celery, lactose).	
Tomato cream soup	Tomato pulp (48%) (origin: EU), water, carrot, tomato paste (4%), potato, onion, cream (milk) (UHT cream 30%, milk proteins), celery stalk, olive oil, sugar, salt, garlic, basil (0.4%), oregano (0.2%), black pepper.	S
Tomato soup	Spring water, tomato paste (13%), rice (11%), onion (4%), carrot (3%), potatoes (2%), rapeseed oil, vegetable broth [vegetable broth base (69.6%) (water, salt, yeast extract, vegetables (carrot, onion, leek, garlic, celery root), sunflower oil, corn starch, concentrated lemon juice, sea salt), water, sugar, corn starch, flavorings, turmeric], salt, parsley, dried vegetables, natural and flavor-enhancing spices.	D
Tomato soup	Water, tomatoes (22.1%), carrot, parsley root, potatoes, celery root, salt, sugar (0.36%), tomato paste, garlic, basil (0.25%), acidity regulator: citric acid, black pepper.	L
Tomato soup	Water, tomato pulp (23%), carrot (5%), rice (4%), chicken meat (3%), cream (milk), modified starch, salt, dried onion, black pepper, parsley leaves, yeast extract, antioxidant: sodium ascorbate.	J
Vegetable soup	Water, potatoes, carrots, cauliflower, leek, parsley root, yellow and green string beans, celery.	B
Vegetable soup	Water, potatoes, carrots, green peas, green beans, cream (milk), celery, onion, leek, butter (milk), vegetable oil (sunflower), modified starch, salt, sugar, natural spices (parsley, pepper, lovage, bay leaf), flavorings.	S
Vegetable soup	Water, vegetables (28%) (carrot, green beans, white cabbage, potatoes, celery, parsley root), pork meat (4%), cream (2%) (milk), wheat flour, salt, modified starch, dill, black pepper, yeast extract, antioxidant: sodium ascorbate.	P
**Instant soup**		
Beetroot soup	Sugar, concentrated beetroot juice (15.9%), maltodextrin, salt, acid (citric acid), flavourings (with wheat), poultry fat, yeast extract, sunflower oil, fermented wheat protein, marjoram, spices.	W
Beetroot soup	Sugar, dried red beet juice concentrate (17.6%), salt, acidity regulator (citric acid), dried vegetables (garlic, onion), flavour enhancers (monosodium glutamate, disodium 5'-ribonucleotides), black pepper (0.4%), rapeseed oil, yeast extract, flavouring.	KU

Beetroot soup	Sugar, maltodextrin, beetroot juice (15%), iodized salt, starch, fructose, garlic (2%), yeast extract, palm fat, acid (citric acid), marjoram, bay leaf, salt, allspice, black pepper.	KN
Broccoli soup	Pea starch, wheat flour, 16.4% wheat flour, sunflower oil, 11.1% vegetables (6.7% broccoli florets, 1.4% broccoli powder, cauliflower, 0.8% spinach), iodized salt, sugar, yeast extract, glucose syrup, whey protein, spices (garlic, nutmeg, black pepper), milk protein, natural plant flavouring, acidifier: citric acid.	W
Button mushroom soup	Corn starch, wheat flour (19.3%), skimmed milk powder (milk) (16.1%), powdered cream (milk) (8.5%), dried champignon mushrooms (8.4%), concentrated mushroom juice (7.1%), salt, flavourings (with wheat), dried onion, poultry fat, parsley leaves, black pepper, acid (citric acid).	W
Cucumber soup	Wheat flour, dried vegetables (pickled cucumber, carrot, celery root, onion, parsnip, garlic, spinach, parsley root), powdered cream (milk), skimmed milk powder (milk), corn starch, salt, sugar, poultry fat, flavourings, citric acid, herbs and spices.	W
Pea soup	Pea flour (64.6%), wheat flour, salt, skimmed milk powder (milk), smoked pork fat, sugar, flavourings (with wheat), dried vegetables (garlic 1.1%, horseradish), yeast extract, herbs, colouring (caramel), spices, smoke flavouring.	W
Pea soup	Water, vegetables (10%) (potatoes, carrots, celery), peas (8%), pork sausage (6%) (pork meat, salt, modified starch, spices, sugar), smoked pork fat, modified starch, wheat flour, salt, black pepper, marjoram, onion, yeast extract, antioxidant: sodium ascorbate.	KU
Sour rye soup	Flour (rye 43.3%, wheat), potatoes (8.7%), iodized salt, flavourings, smoked pork fat (4.5%) (pork fat, antioxidant: rosemary extract), sugar, maltodextrin, whey preparation (milk), mineral potassium salt, acid: citric acid, starch, garlic (1.6%), black pepper, palm fat, hydrolyzed vegetable protein, lactose, marjoram (0.6%), porcini mushrooms (0.6%), mushroom juice concentrate (0.5%), yeast extract, smoke flavouring, salt.	KN
Sour rye soup	Rye flour (55.6%), salt, dried vegetables (7.4%) (onion, garlic), skimmed milk powder (contains lactose), sugar, flavour enhancers (monosodium glutamate, disodium 5'-ribonucleotides), acidity regulator (citric acid), marjoram (1.5%), rapeseed oil, flavourings, smoke flavourings.	KU

Tomato soup	Dried vegetables (35%) (tomato 32.7%, leek, onion, garlic), wheat flour, sugar, salt, corn starch, yeast extract, poultry fat, flavourings (with celery), parsley leaves, spices, citric acid.	W
White borscht	Wheat flour (55.9%), salt, sugar, skimmed milk powder (milk, contains lactose), flavour enhancers (monosodium glutamate, disodium 5'-ribonucleotides), dried vegetables (4.7%) (onion, garlic), acidity regulator (citric acid), thickener (cellulose gum), marjoram, rapeseed oil, black pepper (0.3%), flavourings, smoke flavourings.	KU
White borscht	Wheat flour (44.6%), skimmed milk powder (milk), salt, potato starch, sugar, smoked pork fat, acid (citric acid), flavourings (with wheat), dried vegetables (0.9%) (onion, garlic), marjoram, corn starch, black pepper, smoke flavouring.	W
Wild mushroom soup	Wheat flour, salt, flavour enhancers (monosodium glutamate, disodium 5'-ribonucleotides), mushrooms (2.7%) (champignon, porcini), onion (2.2%), maltodextrin, milk proteins (contain lactose), flavourings, parsley leaves (0.5%), rapeseed oil, caramelized sugar.	KU
Wild mushroom soup	Wheat flour, potato starch, powdered cream (from milk), salt, flavourings (with wheat), dried mushrooms (3.8%) (slippery jack, bay bolete, porcini), poultry fat, dried vegetables (2.4%) (onion, garlic), spices, corn starch, concentrated mushroom juice (1.0%).	W
